# No Association of CALCA Polymorphisms and Aseptic Loosening after Primary Total Hip Arthroplasty

**DOI:** 10.1155/2018/3687415

**Published:** 2018-06-05

**Authors:** Tünay Aydin-Yüce, Gina Kurscheid, Hagen Sjard Bachmann, Thorsten Gehrke, Marcel Dudda, Markus Jäger, Christian Wedemeyer, Max Daniel Kauther

**Affiliations:** ^1^Department of Orthopaedics and Trauma Surgery, University Hospital Essen, Hufelandstraße 55, 45147 Essen, Germany; ^2^Institute of Pharmacology and Toxicology, Witten/Herdecke University, Alfred-Herrhausen-Straße 50, 58455 Witten, Germany; ^3^Department of Joint Surgery, Helios ENDO-Klinik Hamburg, Holstenstraße 2, 22767 Hamburg, Germany; ^4^Department of Orthopaedics and Trauma Surgery, St. Barbara Hospital Gladbeck, Barbarastraße 1, 45964 Gladbeck, Germany

## Abstract

Studies of aseptic loosening showed an influence of calcitonin and *α*-CGRP, both encoded from the calcitonin/*α*-CGRP (CALCA) gene by alternative splicing. The aim of this study was to detect a possible association of the CALCA polymorphisms P1(rs1553005), P2(rs35815751), P3(rs5240), and P4(rs2956) with the time to aseptic loosening after THA. 320 patients suffering from aseptic loosening after primary total hip arthroplasty were genotyped for CALCA-P1 polymorphism and 161 patients for CALCA-P2 and CALCA-P3 polymorphisms and 160 patients for CALCA-P4 polymorphism. CALCA genotypes were determined by polymerase chain reaction and restriction-fragment length polymorphism. The genotype distribution of CALCA-P1 was CC 10%, CT 43%, and 46% TT. CALCA-P2 showed a distribution of 90.7%II, 8.7% ID, and 0.6% DD. The CALCA-P3 genotype distribution was 97.5% TT and 2.5% TC. The CALCA-P4 genotype distribution was 48.1% AA, 40% AT, and 11.9% TT. Significant differences between the CALCA genotypes were not found concerning age at implantation and replantation, BMI, gender, and cementation technique. No associations of the time for aseptic loosening were found. In conclusion, we did not find a significant association of CALCA polymorphisms and the time to aseptic loosening after primary THA in a Western European group.

## 1. Introduction

The prevalence of total hip arthroplasties (THA) and subsequently its revision surgery is increasing mostly due to particle-induced osteolysis (PIO) in the long-term [1]. In this process macrophages get activated by phagocytizing wear particles. They secrete proinflammatory cytokines increasing osteoclastogenesis leading to bone resorption via the induction of the receptor activator of nuclear factor-кB ligand (RANKL) [2–4]. Various studies focused on the genetic background of particle-induced osteolysis (PIO) [5–9]. Still, a reliable marker for detection of patients at high risk of early PIO is still missing.

The CALCA gene is found on chromosome 11p15.2-p15.1 and codes for the neuropeptides alpha-calcitonin gene-related peptide (*α*-CGRP) and calcitonin. It is known for several single nucleotide polymorphisms (SNPs) [10]. CALCA SNPs have been associated with diabetic retinopathy [11], psoriasis vulgaris [12], ovarian cancer [13], and essential hypertension [14]. A pilot study of 87 patients did not find an association of the CALCA-1786T>C (rs117862925) polymorphism (on time to aseptic loosening, while increasing evidence exists for a possible association of CALCA derived neuropeptides and PIO [15]. Alpha-CGRP is a 37-amino acid neuropeptide with multiple physiological affects [16–19]. It plays an important role in neuro-osteogenic interactions with an influence on bone remodeling [20, 21]. Bone tissue is known to contain *α*-CGRP-immunoreactive sensory nerve fibres for downregulating the production of proresorptive cytokines and growth factors and preventing osteoclastogenesis [22, 23]. *α*-CGRP-immunoreactive nerve fibres were found in the periprosthetic membrane and neocapsules of patients with loosed implants, while *α*-CGRP was present in the synovial fluid [21, 24]. Further in vitro [25–27] and in vivo [28] studies stressed its importance in the process of PIO. Calcitonin derives from the parafollicular cells and is known to regulate bone calcium metabolism. Jablonski et al. showed an influence of calcitonin on particle-induced inflammation in THP-1 macrophage-like cells [29]. Furthermore, the substitution of calcitonin in a murine model of calcitonin deficiency significantly reduced PIO [30, 31].

The aim of our study is to answer the question, if there is an association of further calcitonin/*α*-CGRP polymorphisms with the time to aseptic loosening after THA.

## 2. Materials and Methods

Our cohort consisted of 320 Western European patients of German ancestry with aseptic loosening after primary implantation of a THA. These patients were operated at the Helios ENDO-Klinik Hamburg, Germany. We defined strict inclusion and exclusion criteria. Inclusion criteria were clinical, radiological, and intrasurgical diagnosis of aseptic loosening after total hip arthroplasty due to primary osteoarthritis. Exclusion criteria were traumatic loosening, inflammatory diseases, treatment with immunosuppressant, or the suspicion of implant infection, which was diagnosed with microbiological swab analysis. 320 patients were genotyped for CALCA-P1 (rs1553005) polymorphism. Due to loss of samples DNA of 161 patients was genotyped for CALCA-P2 (rs35815751) and CALCA-P3 (rs5240) polymorphisms and 160 patients for CALCA-P4 (rs2956) polymorphism. This study was preformed according to the Declaration of Helsinki after being approved by the Ethics Committee of the University Hospital Essen, Germany.

### 2.1. Experimental Procedures

DNA was extracted from whole blood or buccal swab by using the QIAamp DNA blood mini kit (Qiagen, Hilden, Germany). Polymerase chain reaction (PCR) performed by using Taq DNA polymerase (Sigma-Aldrich, Stockholm, Sweden) under using following PCR conditions: 35 cycles at 94°C for 40 seconds, 56°C for 45 seconds, and 72°C for one minute. The PCR products were run on 1% low melting agarose gels and cut. Ethidium bromide and UV transluminations were used for visualizing the PCR products. Frequencies of identifies polymorphisms were mapping using the restriction enzymes AluI, Acil, PshAI, and BsmAI for separation by agarose-gel electrophoresis and were determined by restriction-fragment length polymorphism (RFLP). Sequence and genomic structure of the CALCA gene were adapted from the GenBank [32]. The CALCA polymorphisms were identified, 2001 (Table 1) [33].

### 2.2. Statistical Analysis

Log-rank test and Kaplan-Meier plots were used to evaluate the relationship between CALCA genotypes, gender, and time to loosening. The Kruskal-Wallis test was applied for comparison of median time to aseptic loosening and the different genotypes. The relation of age, BMI, and CALCA genotypes as prognostic factors for time to aseptic loosening was analysed by univariate and multivariate Cox regression models. We calculated hazard ratios (HR) and 95% CI from these Cox regression models. Continuous variables were compared by ANOVA. Categorical variables were analysed by *χ*^2^ statistics. Differences were considered significant at p<0.05. Statistical analysis was calculated using SPSS 20.0 (SPSS, Chicago, IL, USA).

## 3. Results and Discussion

### 3.1. CALCA Genotypes and Clinical Characteristics

Clinical characteristics and CALCA genotypes distribution in patients with aseptic loosening are given in Tables 2–5. Age at implantation and replantation, BMI, gender, first stem with or without cement, and first cup with or without cement did not show significant differences in the analysed CALCA genotypes.

#### 3.1.1. CALCA-P1 (rs1553005)

Table 2 shows the CALCA-P1 distribution of 320 patients with aseptic loosening after primary THA. There were 34 CC genotype carriers and 139 heterozygous patients and 147 carried the TT genotype. Analysis of the clinical characteristics did not show differences between the genotypes (age at implantation p=0.891; age at replantation p=0.912; gender p=0.817; BMI p=0.648 first cup with cement p=0.329; and first stem with cement p=0.743).

#### 3.1.2. CALCA-P2 (rs35815751)

Table 3 shows the CALCA-P2 distribution of 161 patients with aseptic loosening after primary THA. There were 146 II genotype carriers and 14 heterozygous patients and 1 carried the DD genotype. Analysis of the clinical characteristics showed no statistical significant differences between the genotypes (age at implantation p=0.817; age at replantation p=0.907; gender p=0.802; BMI p=0.587 first cup with cement p=0.205; and first stem with cement p=0.652).

#### 3.1.3. CALCA-P3 (rs5240)

Table 4 displays the CALCA-P3 distribution of 161 patients with aseptic loosening after primary THA. The distribution of the genotypes showed 157 patients with TT and 4 patients with TC genotype. TT genotype had a significant lower BMI than TC genotype. There is no significant correlation in age at implantation (p=0.44), age at replantation (p=0.640), gender (p=0.656), first cup with cement (p=0.126), and first stem with cement (p=0.136).

#### 3.1.4. CALCA-P4 (rs2956)

The CALCA-P4 distribution of 160 patients with aseptic loosening after primary THA showed 77 AA genotype carriers, 64 AT genotypes, and 19 TT genotypes (Table 5). We did not find significant differences between the genotypes for age at implantation (p=0.735), age at replantation (p=0.7429, gender (p=0.117), BMI (p=0.587), first cup with cement (p=0.205), and first stem with cement (p=0.652).

### 3.2. CALCA Genotypes and Time to Aseptic Loosening

**Figure 1:**
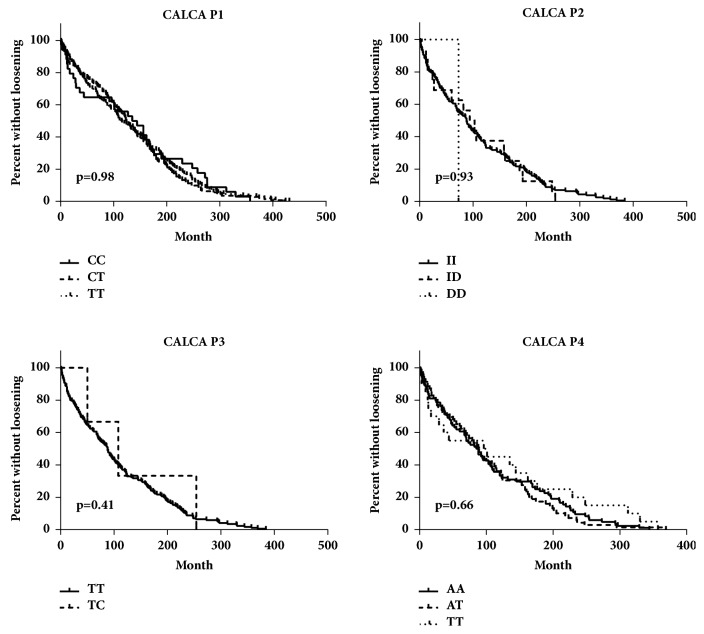
Time to aseptic loosening based on Kaplan-Meier curves for CALCA-P1, CALCA-P2, CALCA-P3, and CALCA-P4 patients.

The correlation of time to aseptic loosening with the CALCA genotype was analysed by using Kaplan-Meier survival curves and Kruskal-Wallis test (Figure 1 and Table 6). No significant difference could be found.

## 4. Discussion

Aseptic loosening after THA is a major problem because of the limited possibilities of multiple replacements. In contrast to septic loosening, only little is known about risk factors influencing aseptic loosening. The incidence of PIO is known to be up to by 2.6% per additional BMI unit higher for male and young patients with sporting activity because of an increasing number of mechanically derived particles [34]. It is known that SNPs influence the incidence of or time to aseptic loosening [5–9]. SNPs of important cytokines in PIO like interleukin-6, tumor necrosis factor-*α*, and transforming growth factor-*β*1 were suggested to predict the time to onset of PIO [35]. A study of the T393C polymorphism (rs7121) in the GNAS1 gene could not find an association with risk for and time to aseptic loosening after THA [36]. XIONG et al. did not find an association of the calcitonin receptor AluI gene polymorphism and bone mineral density [37]. The above-mentioned existing evidence of the influence of the CALCA gene and its gene products [21, 24–31] was the reason to investigate further CALCA polymorphisms in a larger collective.

The aim of our study was to predict the time risk and time to aseptic loosening in association with further CALCA-P1, CALCA-P2, CALCA-P3, and CALCA-P4 polymorphisms as many in vitro and in vivo studies revealed the importance of calcitonin and *α*-CGRP in the complex process of PIO.

This study showed an uneven and independent distribution of the analysed CALCA genotypes. CALCA-P1 CT and TT genotypes were found most often. Only 10.5 % of the patients were CC genotype carriers. The analysis of the CALCA-P2 polymorphism showed a high rate of the II genotype of about 90%. The CALCA P3 TC genotype might have a protective influence on aseptic loosening with an occurrence of 2.5% in this study. The analysis of CALCA P4 shows a high distribution of AA and AT genotypes in patients with aseptic loosening. The time to aseptic loosening did not differ in the analysed genotypes.

In line with this study, Wedemeyer et al. did not find an association of another CALCA polymorphism (rs117862925) in a smaller group [15]. The identification of further genetic risk factors for PIO may result in a specific prophylactic treatment for a better outcome of patients with THA.

## 5. Conclusions

To conclude, we find neither an association with clinical characteristics of the patients nor an association of the analysed calcitonin/*α*-CGRP CALCA-P1, CALCA-P2, CALCA-P3, and CALCA-P4 polymorphisms and the time to aseptic loosening after primary THA in a Western European patient group of German ancestry.

## Figures and Tables

**Table 1:** Localization of the analysed CALCA polymorphisms P1, P2, P3, and P4 in the CALCA genomic structure [33].

**CALCA-**	**SNP rs cluster ID**	**Gen-locus**	**CALCA position**	**forward primer**	**reverse primer**	**product size**
**P1**	rs1553005	g.1210	5′near gene	catttcaaagatgagtacactgcg	gcccaagaaatctgactcca	186 bp

**P2**	rs35815751	g.2919	intron 1	Ccagaagtccactgtgctga	aagggggagaacttttggaa	211 bp

**P3**	rs5240	g.4198	exon 3	Agcctgcactgagtttgctt	gggatccaccttccrgtgta	239 bp

**P4**	rs2956	g.6609	3′near gene	Aaccctgagatcatcaacca	aaagggcaaatacagttcttga	196 bp

**Table 2:** Clinical characteristics and CALCA P1 genotype distribution in patients with aseptic loosening.

	All	*CALCA P1* genotype			p-value
		CC	CT	TT	
*Aseptic loosening*					
n (%)	320 (100)	34 (10.5)	139 (43.0)	147 (45.5)	
Age at implantation (y)	55.0 ± 13.37	55.67 ± 14.7	54.63 ± 12.3	55.24 ± 14.07	0.891
Age at replantation (y)	66.59 ± 12.03	67.12 ± 11.7	66.29 ± 11.7	66.76 ± 12.47	0.912
Gender					
Female (%)	225 (70.3)	25 (11.1)	99 (44)	101 (44.9)	0.817
Male (%)	95 (29.7)	9 (9.5)	40 (42.1)	46 (48.6)	
BMI (kg/m^2^)	26.86 ± 5.0	26.69 ± 5.0	26.28 ± 4.86	27.37 ± 5.3	0.648
First cup with cement (n=294)					
no	107 (36.4)	8 (25)	49 (39.2)	50 (36.5)	0.329
yes	187 (63.6)	24 (75)	76 (60.8)	87 (63.5)	
First stem with cement (n= 292)					
no	105 (36)	10 (9.5)	42 (40)	53 (50.5)	0.743
yes	187 (64)	21(11.2)	80 (42.9)	86 (45.9)	

Data are numbers with percentages given in brackets and numbers with standard deviation, respectively. Categorical variables were analysed by *χ*^2^ statistics. P values were calculated using ANOVA for continuous variables.

**Table 3:** Clinical characteristics and CALCA P2 genotype distribution in patients with aseptic loosening.

	All	*CALCA P2* genotype			p-value
		II	ID	DD	
*Aseptic loosening*					
n (%)	161 (100)	146 (90.7)	14 (8.7)	1 (0.6)	
Age at implantation (y)	57.12 ± 13.7	57.07 ± 14.0	57.0 ± 10.89	66.0 ± 0	0.817
Age at replantation (y)	66.80 ± 12.17	66.72 ± 12.3	67.2 ± 10.6	71.91 ± 0	0.907
Gender					
Female (%)	113 (70.2)	102 (90.3)	10 (8.9)	1 (0.8)	0.802
Male (%)	48 (19.8)	44 (91.7)	4 (8.3)	0	
BMI (kg/m^2^)	26.90 ± 5.0	26.79 ± 5.1	27.77 ± 5.17		0.587
First cup with cement (n=154)					
no	56 (36.4)	52 (92.9)	3 (5.4)	1 (1.7)	0.205
yes	98 (63.6)	87 (88.8)	11 (11.2)	0	
First stem with cement (n= 151)					
no	53 (35.1)	49 (92.5)	4 (7.5)	0	0.652
yes	98 (64.9)	87 (88.8)	10 (10.2)	1 (1)	

Data are numbers with percentages given in brackets and numbers with standard deviation, respectively. Categorical variables were analysed by *χ*^2^ statistics. P values were calculated using ANOVA for continuous variables.

**Table 4:** Clinical characteristics and CALCA-P3 genotype distribution in patients with aseptic loosening.

	All	*CALCA P3* genotype		p-value
		TT	TC	
*Aseptic loosening*				
n (%)	161 (100)	157 (97.5)	4 (2.5)	
Age at implantation (y)	57.12 ± 13.7	57.25 ± 13.8	52.0 ± 12.1	0.44
Age at replantation (y)	66.80 ± 12.17	66.87 ± 12.2	63.97 ± 10.9	0.640
Gender				
Female (%)	113 (70.2)	110 (97.3)	3 (2.7)	0.656
Male (%)	48 (29.8)	47 (97.9)	1 (2.1)	
BMI (kg/m^2^)	26.90 ± 5.08	26.72 ± 5.0	34.27 ± 0.87	0.037*∗*
First cup with cement (n=154)				
no	56 (36.4)	56 (100)	0	0.126
yes	98 (63.6)	94 (95.9)	4 (4.1)	
First stem with cement (n= 151)				
no	53 (35.1)	53 (100)	0	0.136
yes	98 (64.9)	94 (95.9)	4 (4.1)	

Data are numbers with percentages given in brackets and numbers with standard deviation, respectively. Categorical variables were analysed by *χ*^2^ statistics. P values were calculated using ANOVA for continuous variables. Significant differences are marked with *∗* for p<0.05.

**Table 5:** Clinical characteristics and CALCA-P4 genotype distribution in patients with aseptic loosening.

	All	*CALCA P4* genotype			p-value
		AA	AT	TT	
*Aseptic loosening*					
n (%)	160 (100)	77 (48.1)	64 (40)	19 (11.9)	
Age at implantation (y)	57.16 ± 13.7	57.81 ± 13.3	57.17 ± 12.6	54.52 ± 18.88	0.735
Age at replantation (y)	66.76 ± 12.2	67.48 ± 12.4	66.31 ± 11.7	65.36 ± 12.9	0.742
Gender					
Female (%)	112 (70)	48 (42.9)	50 (44.6)	14 (12.5)	0.117
Male (%)	48 (30)	29 (60.5)	14 (29.1)	5 (10.4)	
BMI (kg/m^2^)	26.90 ± 5.11	27.46 ± 5.2	26.24 ± 5.0	26.69 ± 5.04	0.590
First cup with cement (n=153)					
no	56 (36.6)	28 (50)	23 (41.1)	5 (8.9)	0.598
yes	97 (63.4)	47 (48.5)	36 (37.1)	14 (14.4)	
First stem with cement (n= 150)					
no	53 (35.3)	30 (56.6)	17 (32.1)	6 (11.3)	0.546
yes	97 (64.7)	46 (47.4)	39 (40.2)	12 (12.4)	

Data are numbers with percentages given in brackets and numbers with standard deviation, respectively. Categorical variables were analysed by *χ*^2^ statistics. P values were calculated using ANOVA for continuous variables.

**Table 6:** Median time to aseptic loosening according to gender and CALCA genotype.

n (%)	All 315	CALCA P1- genotype			p-value
		CC 34 (10.8)	CT 136 (43.2)	TT 145 (46.0)	
MTAL, mo (range)	128 (1-431)	139 (1-357)	128 (1-424)	120 (1-431)	0.971

	All	CALCA P2- genotype			p-value 0.77
		II	ID		DD

n (%)	159	144 (90.6)	14 (8.8)		1 (0.6)
MTAL, mo (range)	92 (1-384)	90 (1-384)	104 (15-254)		73 (73-73)

	All	CALCA P3- genotype			
		TT	TC		p-value

n (%)	159 (100)	155 (97.5)	4 (2.5)		
MTAL, mo (range)	92 (1-384)	92 (1-384)	138 (50-254)		0.339

	All	CALCA P4- genotype			p-value 0.976
		AA	AT		TT

n (%)	158	76 (48.1)	63 (39.9)		19 (12.0)
MTAL, mo (range)	92 (1-384)	88 (1-344)	92 (1-369)		101 (1-357)

MTAL, median time to aseptic loosening. Data are numbers with percentages given in brackets and medians with ranges given in brackets, respectively. P values were calculated using Kruskal-Wallis test for nonparametric variables. *∗*Jonckheere-Terpstra test for linear comparison of nonparametric variables, p=0.160.

## Data Availability

Data of this study is available on request to the corresponding author after being approved by the Ethics Committee of the University Hospital Essen, Germany.
